# DGM-TOP: automatic identification of the critical boundaries in atrial tachycardia

**DOI:** 10.3389/fphys.2025.1563807

**Published:** 2025-05-27

**Authors:** Robin Van Den Abeele, Sander Hendrickx, Niels Carlier, Eike M. Wülfers, Arthur Santos Bezerra, Bjorn Verstraeten, Sebastiaan Lootens, Karel Desplenter, Arstanbek Okenov, Timur Nezlobinsky, Annika Haas, Armin Luik, Sebastien Knecht, Mattias Duytschaever, Nele Vandersickel

**Affiliations:** ^1^ Department of Physics and Astronomy, Ghent University, Ghent, Belgium; ^2^ Department of Cardiology, Städtisches Klinikum Karlsruhe, Karlsruhe, Germany; ^3^ Department of Cardiology, AZ Sint-Jan, Bruges, Belgium

**Keywords:** atrial tachycardia (AT), topology, simulation - computers, ablation < electrophysiology, clinical analysis

## Abstract

**Introduction:**

In the latest research on topology in cardiac arrhythmia, it was demonstrated through a fundamental mathematical principle called the index theorem that reentry based atrial tachycardias (AT) are maintained by pairs of counter-rotating waves that are either complete or near-complete rotations. Each wave is centered around a different anatomical object that exhibits a non-zero index/topological charge, called a critical boundary. Interconnecting both critical boundaries with an ablation line terminates the tachycardia.

**Methods:**

This research focuses on the specific algorithms for calculating the index/topological charge of each anatomical boundary, called DGM-TOP. The algorithm used analyzes the electroanatomical map of the patient, extracting the nodes at each boundary. The index is then calculated for each boundary by sequentially summing the differences in local activation time and normalizing by the cycle length. Boundaries with a non-zero index are identified as critical boundaries.

**Results and discussion:**

Using this method, pairs of critical boundaries were consistently detected in 100% of the 578 *in silico* and 100% of the 24 clinical ATs. Adhering to the previously described index theorem. Additionally, ablation results in both datasets show that termination of AT is only possible by interconnecting both critical boundaries. This outcome highlights the importance of detecting the critical boundaries before deciding on the correct ablation line, as any ablation line that does not connect both critical boundaries is unable to terminate the AT. Moreover, in the case of incorrect ablation, the BCL-algorithm was proposed to estimate the increase in tachycardia cycle length. However, only moderate correlation $\left(r^{2}=0.62\right)$ is observed for simulations, indicating a refinement of this BCL-algorithm is necessary in addition to a larger clinical dataset.

## 1 Introduction

Until recently, it was assumed that reentry-based atrial tachycardia (AT) is mostly driven by a single reentry loop (Markowitz et al., 2019; Kuroda et al., 2021; Vlachos et al., 2021), although dual or triple loops have also been reported (Kuroda et al., 2021; Takigawa et al., 2018). It was also widely accepted that entrainment mapping could find the driving reentry circuit(s) (Vlachos et al., 2021; Stevenson et al., 1995; Strisciuglio et al., 2019; Jaïs et al., 2009). The other circuits were sometimes called passive or bystander circuits (Strisciuglio et al., 2019; Pathik et al., 2017) and were not considered ablation targets.

However, previous research (Duytschaever et al., 2024), demonstrated reentry loops consistently occurring in pairs. This discovery allowed us to establish a comprehensive framework for understanding the mechanisms underlying reentry-based AT. Consequently, a novel ablation strategy was proposed based on targeting both reentry loops, potentially transforming current ablation therapy. Concurrently, Santucci et al. (2023) provided empirical support for this concept, further validating its significance.

The Index theorem requires an understanding of the topology of the left atrium (LA) and right atrium (RA). Topologically, the LA and RA can be deformed separately into a spherical surfaces with a number of distinct boundaries. The natural boundaries of the LA are the mitral valve (MV), left pulmonary vein (LPV), and right pulmonary veins (RPV), while the RA is defined by the tricuspid valve (TV), inferior vena cava (IVC), and superior vena cava (SVC). Scarring and conduction block lines form additional boundaries (Markowitz et al., 2019), further transforming the topology of the atria. Importantly, conduction block lines connecting different boundaries, such as those resulting from ablation procedures, merge separate boundaries into a single unified boundary.

The core of the paired loops is rooted in the index theorem (Winfree and Tyson, 1988; Davidsen et al., 2004; Mermin, 1979). This theorem states that on a closed surface with a finite number of boundaries, the sum of topological charge (TC), or phase indexes (I) , must equate to zero (ΣI=0) . This topological charge of an area enclosed by a circumference C is computed via a contour integral in counter-clockwise (CCW) direction:

$$I=\frac{1}{2\pi }\oint_{C}\vec{\nabla }\phi \cdot \vec{d\ell }$$ (1)

here, $\vec{\nabla }\phi$ represents the spatial phase gradient, and $\vec{d\ell }$ is tangent to the circumference. In AT scenarios, this normalized phase ϕ/2π is interpreted as the local activation time (LAT), normalized by the tachycardia cycle length (TCL). As such, Equation 1 can be redefined as:

$$I=\frac{1}{\text{TCL}}\oint_{C}\vec{\nabla }\text{LAT}\cdot \vec{d\ell }$$ (2)

As such, a phase index of −1 or +1 indicates clockwise (CW) or counterclockwise (CCW) rotation, respectively. Therefore, during AT, boundaries with non-zero topological charge are identified as the critical boundary (CB)s, a term coined by Santucci et al. (2023). The remaining boundaries, exhibiting index 0, are termed non-critical boundary (NCB)s. Interconnecting both CBs with an ablation line at their shared isthmus effectively terminates the AT, irrespective of the total number of boundaries. However, ablation from a CB to an NCB will reduce the number of boundaries without terminating the AT. The latter ablation will result in one of two outcomes. Either the TCL remains constant when a complete rotation is not ablated, or the TCL prolongs when all complete rotations are ablated, but not all incomplete rotations.

In the previous study (Duytschaever et al., 2024), the index of a given boundary was determined either visually or based on the ablation response. Therefore, automating the detection of CBs is a critical next step in the analysis and treatment of cardiac arrhythmias. In this paper, we will first present a computer algorithm designed to automatically calculate the index based on LAT data surrounding a boundary and to identify the type of boundary (CB versus NCB).

Additionally, as described by Duytschaever et al. (2024), the prolongation of the TCL after incorrect ablation can be predicted using entrainment mapping values around the CBs. However, entrainment mapping can be both time-consuming and challenging (Tung, 2017), as the process can inadvertently interrupt AT, complicating the procedure (Barbhaiya et al., 2015). Therefore, we also propose a novel method to predict the TCL based on the absolute revolution time of each CB, referred to as the boundary cycle length (BCL). This BCL represents the natural cycle length of a complete reentry around its specific boundary when a complete rotation is sustained. We hypothesize that the shortest BCL from any CB that is not ablated will determine the TCL of any given AT after incorrect ablation. If such a correlation is established, this means that clinical electrophysiologist could forgo the need for entrainment mapping if favor of the BCL algorithm for the identification of the dominant and passive driver and speed up the procedure.

In this study, these two algorithms will be demonstrated on two different datasets: first on a set of 578 simulated AT cases, then on a clinical set of 24 AT cases recorded with CARTO (Biosense Webster) in AZ Sint-Jan in Brugge and RHYTHMIA (Boston Scientific) in the Städische Klinikum in Karlsruhe.

## 2 Methods

### 2.1 Computer simulations

Using the OpenCARP simulator (Plank et al., 2021), an open-source cardiac electrophysiology platform, 578 distinct simulations were conducted to model atrial tachycardias (ATs) on a spherical mesh. This methodology closely follows the approach described in Duytschaever et al. (2024).

#### 2.1.1 Cell model

Given the atrial-like characteristics of the substrate, the Courtemanche-Ramirez-Nattel model (Courtemanche et al., 1998) was selected. To more easily facilitate reentry, the model was modified by reducing the L-type calcium current $I_{\mathrm{CaL}}$ by 70% (Yue et al., 1997) to shorten the action potential. Additionally, the rapid delayed rectifier potassium current $I_{Kr}$ by 70% was increased to further shorten the action potential without destabilizing the arrhythmia (Henry, 2022). This modified model was then pre-paced at a 250 ms interval (S1-S1) for a duration of 50 s to achieve a steady-state condition.

#### 2.1.2 Substrate

A triangulated spherical surface was created with a radius of 20 mm, a mean edge length of 200 μ m, and conduction velocity of 0.4 mm/ms. On this mesh, variety was created by adding non-conductive boundaries in random locations and a random diameter (between 9 and 13 mm). As such, 200 meshes were created with 2 boundaries, 33 with 3 boundaries, and 6 with 4 boundaries. Subsequently, for meshes with 3 and 4 boundaries, one slow conductive zone (SCZ) was added in the area between each possible combination of boundaries. These SCZs had a conduction velocity of 0.2 mm/ms and expanded in width as they approached the central region between the boundaries, increasing by 1 mm in width for every 1.6 mm of distance away from a boundary. The addition of the SCZ resulted in 200 substrates with 2 boundaries, 99 with 3 boundaries, and 36 with 4 boundaries, for a total of 335 unique meshes. One example of a 3-boundary substrate can be seen in Supplementary Figure S1 on the left.

Of note: a conduction velocity of 0.4 mm/ms was chosen to facilitate the induction of stable reentry. While this is lower than typical values reported for non-fibrotic atrial tissue, it remains within a range that permits reentry around anatomical structures. Moreover, using a more physiological conduction velocity of 0.7 mm/ms is unlikely to affect the study’s results.

#### 2.1.3 Reentry induction

AT was induced by pacing (S1-S1: 250 ms) along a path parallel to and 4 mm away from a temporary line of block (LOB) connecting two holes via the shortest path between their perimeters. 200 ms after the final paced beat, the LOB was removed, thereby initiating reentry. For the 2-boundary model, reentry was induced between the two boundaries. In the 3- and 4-boundary models, reentry was induced between any combination of boundaries without a SCZ (two potential sites for the 3-boundary model and five for the 4-boundary model). As such, a total of 578 unique simulations were created: 200 (=200×1×1) 2-boundary simulations, 198 (=33×3×2) 3-boundary simulations, and 180 (=6×6×5) 4-boundary simulations.

The TCL of the AT was determined by calculating the median of the temporal intervals between the last 2 activations of each node.

#### 2.1.4 Ablation

For each simulation, separate instances were created to observe the TCL response to virtual ablation lines. Each ablation consists of a 4 mm thick line of non-excitable tissue connecting two boundaries along the shortest possible path. An example of an ablation can be seen in Supplementary Figure S1 in the right panel. Of note: the whole ablation line was paced at once, not mimicking the clinical point-by-point lesions.

### 2.2 Clinical data

For clinical testing, 65 left-atrial AT cases were collected from patients who underwent RF ablation. To ensure data quality and consistency, the following exclusion criteria were applied:

• The TCL before and after ablation was known.
• The locations of all anatomical boundaries were known.
• The official diagnosis of the case was known and confirmed through ablation. Additionally, the sequence of ablation lines with corresponding ablation response was recorded.
• The electroanatomical map was of sufficient quality, meaning that the minimal local density at each point in the mesh must be greater than 0.004 measurement points/ ${\text{mm}}^{2}$ .

By sequentially applying these criteria, we obtained 65 cases where the TCL was known. Among them, the anatomical boundaries were fully confirmed in 60 cases. From these 60, only 34 had a completely recorded ablation sequence. Finally, of these 34 AT cases, 24 met the required quality standards.

Of the 24 left-atrial AT cases, 16 were recorded at AZ Sint-Jan Hospital in Brugge and 8 at Städtische Klinikum in Karlsruhe between November 2015 and February 2023. The TCL ranged from 200 ms to 300 ms. Among these cases, 21 exhibited three anatomical boundaries, while the remaining three had four boundaries.

For each clinical activation map, in consensus with the electrophysiologist, the locations of the anatomical boundaries were determined and subsequently removed from the mesh. These areas include anatomical objects, scar tissue and lines of block.

### 2.3 DGM-TOP

Directed Graph Mapping, or DGM, is a software framework that uses network theory to analyze cardiac mapping data (Vandersickel et al., 2019; Van Nieuwenhuyse et al., 2020; Van Nieuwenhuyse et al., 2021a; Van Nieuwenhuyse et al., 2021b; Hawson et al., 2022; Lootens et al., 2024; Vandersickel et al., 2024). For this study, a new module was introduced to DGM called DGM-TOP, where ‘TOP’ represents topology. This algorithm is able to determine whether the located boundaries are critical or non-critical by calculation the TC inherent to each anatomical boundary. In addition, this module allows for the calculation of the BCL from which the TCL after incorrect ablation can be estimated.

#### 2.3.1 Index calculation

This section describes the algorithm used to compute the TC index along each boundary in both simulated and clinical cases.

For simulations, the LAT-map was obtained by extracting the first LAT after 3,000 ms after stabilization. No further preprocessing was needed as the simulations did not contain any noise.

**FIGURE 1:**
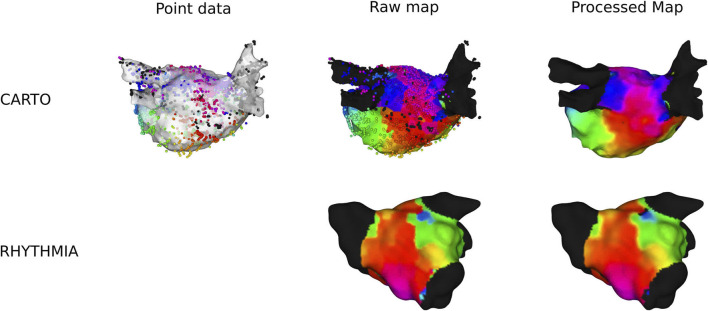
Overview of preprocessing of a clinical anatomical map for CARTO- and RHYTHMIA-recorded maps.

Exported electroanatomical maps from CARTO lack the proprietary preprocessing. Therefore, a custom LAT-map was made using the measurement points, explained as follows. First, all measurements points were projected to the closest location on the anatomical mesh. Second, each mesh point was assigned the LAT-value of the closest measurement point, creating a Voronoi-like raw LAT-map. Finally, the Voronoi-mesh was filtered by taking the average LAT in a radius of 3 mm, while taking into account the periodicity of the activation time to ensure a smoother colormap. However, some blocked lines were still present in the electroanatomical mesh. Therefore, a block-line filter was applied: if the average LAT-deviation to the mean was larger than the empirically determined value of one-sixth the TCL, the point was determined to be ambiguous and was removed from the mesh. A visual representation of this pipeline can be seen in Figure 1.

In RHYTHMIA-recorded ATs, preprocessing was already applied on the export of the electroanatomical map. Therefore, only the average-filter with the block-line filter was applied. An example is shown in Figure 1.

**FIGURE 2:**
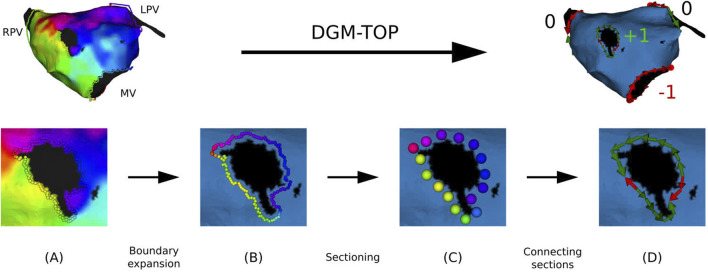
Overview of preprocessing of a clinical anatomical map. **(A)**: extraction of the points at the boundaries. **(B)** Boundary is expanded up to the first order neighbors. **(C)** Points are divided into sections. **(D)** Each section is directed along the conduction vector.

From the preprocessed meshes, the boundaries were identified and expanded, avoiding any incorrect interpolation at the edge and smoothing out the shape of the boundary. Subsequently, the boundary was divided into a certain number of sections, each section containing 4 points. Finally, each section was then summarized by a super-node located at the geometric center of the section and assigned the mean LAT of the retained points ( ${\text{LAT}}_{i}$ ). All steps are illustrated in Figure 2.

Due to the discrete nature of the boundary, the continuous integral defining TC (Equation 2) was approximated as a Riemann sum over the sections in the CCW direction. As a result, the integral was transformed into a summation over sequential sections, with the LAT gradient term replaced by the LAT difference ( $\Delta {\text{LAT}}_{i}$ ) between neighboring sections (Equation 4), leading to the following expression:

$$\text{TC}=\frac{1}{\text{TCL}}\underset{S_{i}}{\sum }\Delta {\text{LAT}}_{i}$$ (3)

$$\Delta {\text{LAT}}_{i}={\text{LAT}}_{i+1}-{\text{LAT}}_{i}$$ (4)

**FIGURE 3:**
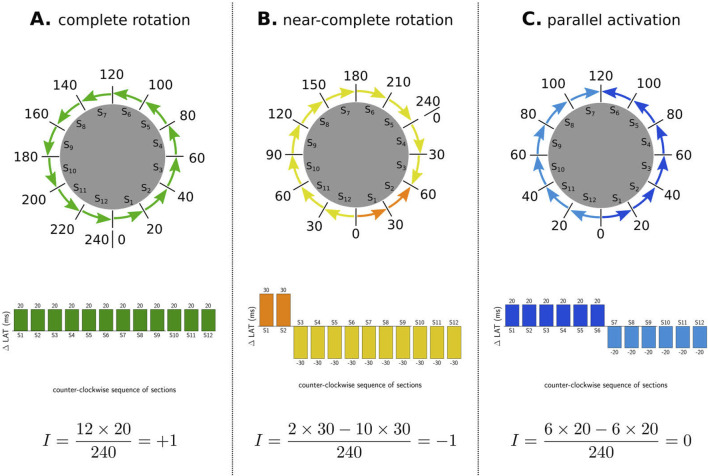
Illustration of index calculation for three different activation patterns. The boundaries were divided into 12 sections, and the $\Delta {\text{LAT}}_{i}$ for each section was recorded in the histogram below. The index was calculated by summing the values of all bars in the histogram. **(A)** Complete rotation pattern yielded an index of +1, characteristic of a CB. **(B)** Near-complete rotation pattern resulted in an index of −1, also indicative of a CB. **(C)** Parallel activation pattern produced an index of 0, characteristic of an NCB.

In general, 3 different types of activation pattern were observed: complete rotation, near-complete rotation, and parallel activation (Duytschaever et al., 2024). In Figure 3, representative examples of each type are shown (TCL = 240 ms).

First, in the case of a complete rotation, the total revolution around the boundary equals the TCL (=240 ms in the CCW direction in example A). Using Equation 3, the TC equals +1 .

Second, in the case of near-complete rotation, the wave propagates in one direction for a duration longer than the TCL (e.g., 300 ms in example B) and in reverse direction for the additional time (e.g., 60 ms). Using Equation 3, this pattern results in an index of −1 for the boundary.

Third, in the case of parallel activation, as illustrated in example C, the wave propagates equally in both the CW and CCW directions (120 ms each). According to Equation 3, this results in an index of 0 for the boundary.

##### 2.3.1.1 Validation

The algorithm was validated based on the ablation line and the ablation response of the AT. First, DGM-TOP identified the locations of both CBs. The algorithm was considered correct if the clinical AT was successfully terminated by ablating between the two CBs. Additionally, if the ablation line was applied between a CB and an NCB, DGM-TOP was deemed correct if the ablation response resulted in a reentry around the non-ablated CB.

#### 2.3.2 Boundary cycle length

The BCL was calculated similarly to the index or topological charge described in the previous section, but instead of summing the LAT differences, the absolute values of these differences were summed, as shown in Equation 5. We hypothesize that this would represent the natural revolution time of the boundary.

$$\text{BCL}=\frac{1}{\text{TCL}}\underset{S_{i}}{\sum }|\Delta \text{LAT}|$$ (5)

The BCL was computed for both the simulations and clinical cases and analyzed the linear correlation of the TCL after ablation to the BCL of the CBs that are not ablated. The $r^{2}$ correlation between both values was calculated and the p-value was determined by means of a Wald-test.

## 3 Results

### 3.1 Simulations

In total, 578 unique simulations were conducted on a spherical mesh with 2 (200), 3 (198), or 4 (180) boundaries. The 2-boundary models had a median cycle length of 231 ms with an IQR from 220 ms to 241 ms (Supplementary Figure S2). The 3-boundary models had a median cycle length of 201 ms with an IQR from 192 ms to 207 ms (Supplementary Figure S3). The 4-boundary models had a median cycle length of 203 ms with an IQR from 195 ms to 208 ms (Supplementary Figure S4).

In all simulations, a total of 1714 boundaries were applied. In this dataset 1156 boundaries were identified as CBs, while the remaining 558 boundaries were NCBs with index 0. More specifically, in each of the 578 unique simulations, 1 boundary was identified with an index of +1 and 1 boundary with an index of −1. The remaining boundaries in the simulation had an index of 0. This identification was based on the ablation response of the simulation: The boundaries involved with the terminating ablation line were labeled as CB.

#### 3.1.1 Index calculation

**FIGURE 4:**
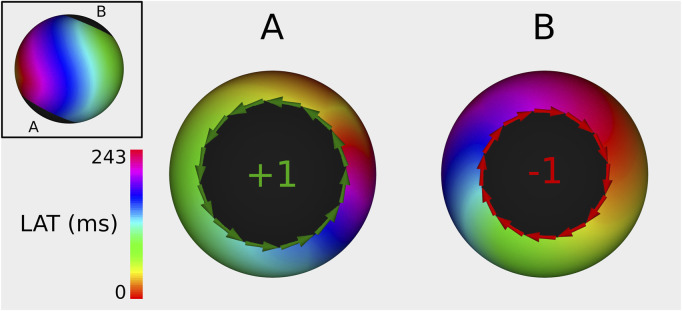
Example of index calculation on a 2B simulation with a TCL of 243 ms from 2 different viewpoints (one for each boundary). Boundary **(A)** has an index of +1, while boundary **(B)** has an index of −1. Therefore, the sum of all TC totals 0. The ablation line between boundary **(A,B)** terminated the AT.

**FIGURE 5:**
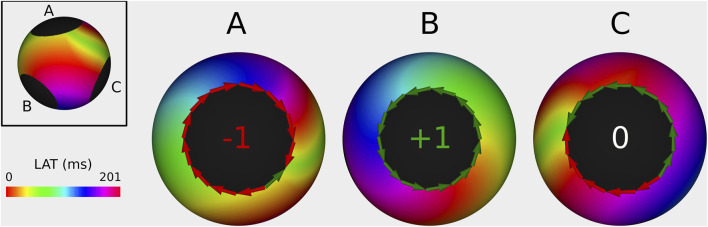
Example of index calculation on a 3B simulation with a TCL of 201 ms from 3 different viewpoints (one for each boundary). Boundary **(A)** has an index of −1, boundary **(B)** has an index of +1, and the boundary **(C)** has an index of 0. Therefore the sum of all TC totals 0. The ablation line between boundary **(A,B)** terminated the AT.

**FIGURE 6:**
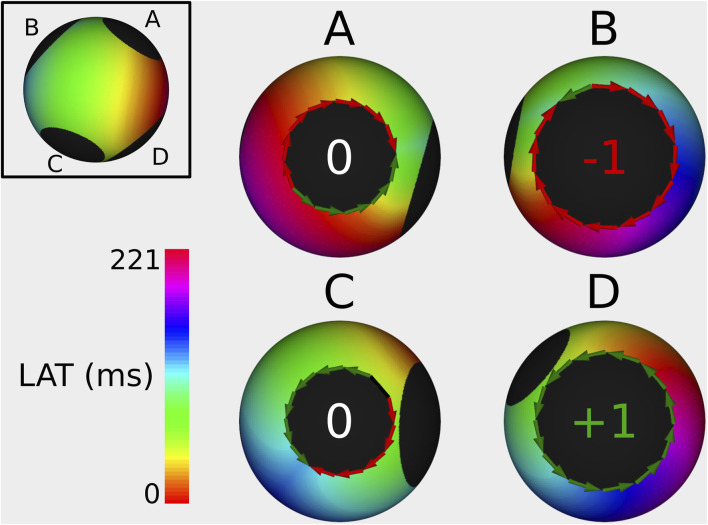
Example of index calculation on a 4B simulation with a TCL of 221 ms from 4 different viewpoints (one for each boundary). Boundary **(A)** has an index of 0, boundary **(B)** has an index of −1, the boundary **(C)** has an index of 0, and boundary **(D)** has an index of +1. The sum of all TC is 0. The ablation line between boundary **(B,D)** terminated the AT.

In 100% of the cases, the index calculation correctly identified the CBs and the NCBs. First, a representative example of an index calculation for a 2-boundary model is shown in Figure 4. In this simulation, the TCL was equal to 243 ms, with one boundary having an index of +1, while the other boundary had an index of −1. The sum of the indexes was therefore zero. All other simulations were analogous. Figures 5, 6 show similar examples of 3- and 4-boundary models.

Determining the distribution of complete and incomplete rotations was a matter of symmetry and could be identified by analyzing the prolongation of TCL after ablation. If the maximal prolongation after one of the possible ablations was more than an arbitrary threshold of 5 ms, the simulation was labeled as “asymmetrical”, meaning an incomplete rotation was present alongside the TCL-determining complete rotation. Otherwise, if the maximal prolongation was lower than 5 ms, the case was labeled “symmetrical”, meaning that 2 complete rotations were present. Inherently, this protocol could only be applied to 3- and 4-boundary simulations. As such, 192 (97%) out of 198 3-boundary simulations and 169 (94%) out of 180 4-boundary simulations would be labeled as asymmetric.

Ablation results between each possible pair of boundaries showed consistent termination of the AT when interconnecting the CB pair as revealed by DGM-TOP. Any other pair-wise ablation caused either a prolongation in TCL or no change in TCL. By our criteria, this makes DGM-TOP 100% accurate in identification of CBs concerning simulated ATs.

#### 3.1.2 Boundary cycle length

In this second step, the predictive power of the BCL algorithm was investigated with respect to the TCL after performing virtual ablation. For this purpose, we correlated for each simulation the maximal TCL after the creation of the ablation line with the maximal BCL of any CB that was not ablated. Per protocol, only 3B and 4B cases were analyzed, as 2B cases only have one possible ablation line, which always connects the CB pair, thus terminating the AT.

Fitting a linear function through the 378 data points, using linear regression, a positive correlation was achieved with a slope of 0.681±0.028 and an intercept of 53.6±6.7 ms, with a coefficient of determination $\left(r^{2}\right)$ of 0.62 and a p-value of $2.20\cdot 10^{-80}$ as calculated by the Wald test.

To rule out selection bias as a potential cause of this correlation, we also compared the TCL before ablation to the TCL after ablation (Supplementary Figure S5). For this relationship, a slope of 0.156±0.032 was obtained, with an intercept of 164.9±7.9 , an $r^{2}$ of 0.058, and a p-value of $2.31\cdot 10^{-5}$ , indicating a lack of correlation between the TCL before and after ablation.

### 3.2 Clinical cases

#### 3.2.1 Index calculation

A total of 24 clinical cases of AT were investigated in depth using the DGM-TOP algorithm. 21 ATs contained 3 boundaries, while the 3 other ATs had 4 boundaries, for a total of 75 boundaries. After the boundaries were determined, the index/topological charge was calculated using the DGM-TOP algorithm. In each AT, 2 CBs were found, resulting in a total of 48 CBs. The remaining 27 boundaries were labeled NCB as they had an index equal to 0. Additionally, in each map, the total index was 0 as predicted by the index theorem.

More general clinical data for each AT can be seen in Table 1, with data relevant to the validation criteria in Table 2.

**TABLE 1:** Data for all clinical ATs, including the mapping system with the catheter, the tachycardia cycle length of the AT, the number of anatomical boundaries in the left atrium, the clinical diagnosis at the time of mapping and mesh density.

AT	Mapping system	Catheter	TCL	# boundaries	Clinical diagnosis	Minimal point density (points/ $mm^{2}$ )
${AT}_{1}$	CARTO	Pentarray	250	3	RPV	0.0086
${AT}_{2}$	CARTO	Pentarray	200	3	LPV	0.0062
${AT}_{3}$	CARTO	Point-by-point	260	4	MV and scar	0.0056
${AT}_{4}$	CARTO	Pentarray	265	3	RPV and MV	0.0094
${AT}_{5}$	RHYTHMIA	Orion	260	3	RPV and LPV	0.0501
${AT}_{6}$	RHYTHMIA	Orion	275	3	MV and LPV	0.0434
${AT}_{7}$	CARTO	Pentarray	260	3	MV and LPV	0.0100
${AT}_{8}$	CARTO	Pentarray	220	3	MV	0.0118
${AT}_{9}$	CARTO	Pentarray	236	3	MV	0.0050
${AT}_{10}$	CARTO	Point-by-point	230	3	MV and LPV	0.0044
${AT}_{11}$	CARTO	Pentarray	260	3	MV and LPV	0.0103
${AT}_{12}$	CARTO	Pentarray	200	3	MV	0.0048
${AT}_{13}$	RHYTHMIA	Orion	210	3	RPV and LPV	0.0419
${AT}_{14}$	CARTO	Pentarray	300	3	RPV	0.0081
${AT}_{15}$	RHYTHMIA	Orion	270	3	RPV	0.0680
${AT}_{16}$	RHYTHMIA	Orion	250	3	MV	0.0294
${AT}_{17}$	RHYTHMIA	Orion	260	3	RPV and LPV	0.0205
${AT}_{18}$	CARTO	Pentarray	240	3	LPV	0.0125
${AT}_{19}$	RHYTHMIA	Orion	240	3	LPV	0.0417
${AT}_{20}$	RHYTHMIA	Orion	240	3	LPV	0.0396
${AT}_{21}$	CARTO	Pentarray	240	3	RPV	0.0051
${AT}_{22}$	CARTO	Pentarray	290	4	MV and scar	0.0127
${AT}_{23}$	CARTO	Point-by-point	240	3	RPV and LPV	0.0070
${AT}_{24}$	CARTO	Pentarray	300	4	MV and scar	0.0065

**TABLE 2:** Data for all clinical ATs, including the diagnosis of the AT as of time of recording, the diagnosis by calculation of the CBs, the ablations line that was induced and the response of the AT after placing the ablation line. This response was either termination of the AT or a transition to a second AT.

AT	Clinical diagnosis	Critical boundaries	Ablation line	Ablation response
${AT}_{1}$	RPV	RPV and MV	LPV to RPV	MV
${AT}_{2}$	LPV	LPV and MV	LPV to RPV	MV
${AT}_{3}$	MV and scar	MV and scar	MV to scar	Termination
${AT}_{4}$	RPV and MV	RPV and MV	LPV to RPV	MV
${AT}_{5}$	RPV and LPV	RPV and LPV	RPV to LPV	Termination
${AT}_{6}$	MV and LPV	MV and LPV	MV to LPV	Termination
${AT}_{7}$	MV and LPV	MV and LPV	MV to LPV	Termination
${AT}_{8}$	MV	MV and LPV	MV to LPV	Termination
${AT}_{9}$	MV	MV and LPV	MV to LPV	Termination
${AT}_{10}$	MV and LPV	MV and LPV	MV to LPV	Termination
${AT}_{11}$	MV and LPV	MV and LPV	LPV to RPV	MV
${AT}_{12}$	MV	MV and RPV	MV to LPV to RPV	Termination
${AT}_{13}$	RPV and LPV	RPV and LPV	LPV to RPV	Termination
${AT}_{14}$	RPV	RPV and MV	LPV to RPV	MV
${AT}_{15}$	RPV	RPV and LV	LPV to RPV	Termination
${AT}_{16}$	MV	MV and LPV	LPV to MV	Termination
${AT}_{17}$	RPV and LPV	RPV and LPV	LPV to RPV	Termination
${AT}_{18}$	LPV	LPV and MV	LPV to RPV	MV
${AT}_{19}$	LPV	LPV and MV	LPV to RPV	MV
${AT}_{20}$	LPV	LPV and RPV	LPV to RPV	Termination
${AT}_{21}$	RPV	RPV and LPV	RPV to LPV	Termination
${AT}_{22}$	MV and scar	MV and scar	MV to scar	Termination
${AT}_{23}$	RPV and LPV	RPV and LPV	LPV to RPV	Termination
${AT}_{24}$	MV and scar	MV and scar	LPV to scar	MV and LPV+scar

To summarize, 16 clinical ATs were terminated after ablation by interconnecting the CB-pair as calculated by DGM-TOP. In the remaining 8 cases, The ablation did not terminate the AT. However, all ablation responses show the non-ablated CB as a main reentrant driver. Therefore, DGM-TOP has a 100% accuracy according to the validation criteria.

For 3 ATs, the calculation of the topological charge is shown in more detail below.

**FIGURE 7:**
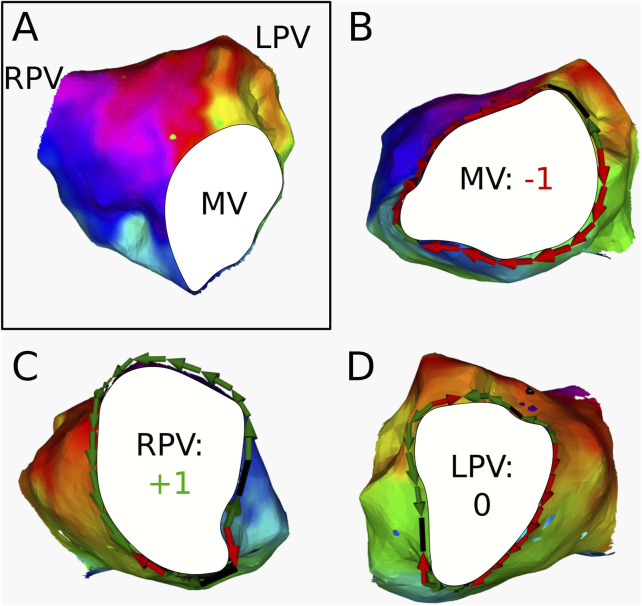
**(A)** Electroanatomical map of ${\text{AT}}_{1}$ in anterior-posterior (AP) view. The cyclical colors on the figure represent the LAT of the map. The boundaries are colored in white. **(B)** Conduction around the MV. The index was determined to be −1 **(C)** Conduction around the RPV. The index was calculated to be +1 **(D)** Conduction around the LPV. The index was determined to be 0. This AT was clinically ablated with a line from the LPV to the RPV, resulting in a second AT with reentry around the MV.

${AT}_{1}$ (Figure 7) was labeled as an RPV-reentry with a period of 250 ms by the electrophysiologist. The AT was determined to contain 3 boundaries: the RPV, LPV, and MV. DGM-TOP confirmed the original diagnosis as the RPV is seen as a CB in Figure 7C. Furthermore, MV (Figure 7B) was labeled as CB. This was confirmed through ablation as the AT converted to an MV-reentry with a period of 285 ms after connecting the RPV and LPV.

**FIGURE 8:**
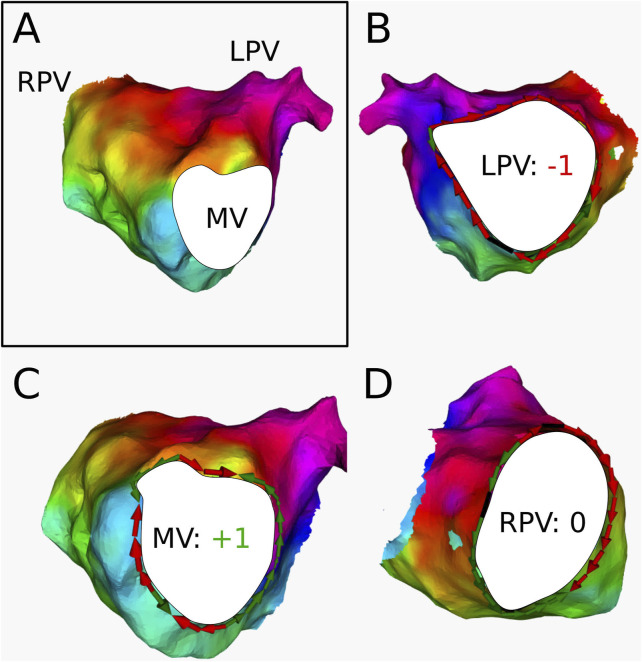
**(A)** Electroanatomical map of ${\text{AT}}_{2}$ in anterior-posterior (AP) view. The cyclical colors on the figure represent the LAT of the map. The boundaries are colored in white. **(B)** Conduction around the LPV. The index was determined to be −1 **(C)** Conduction around the MV. The index was determined to be +1 **(D)** Conduction around the RPV. The index was determined to be 0. This AT was clinically ablated with a line from the LPV to the RPV, resulting in a second AT with reentry around the MV.

${AT}_{2}$ (Figure 8) was diagnosed as an LPV-reentry with a period of 200 ms by the electrophysiologist. The AT was determined to contain 3 boundaries: the RPV, LPV, and MV. After ablation of the roof (RPV to LPV), the AT transitioned to an MV-reentry with a period of 230 ms. DGM-TOP determined the LPV and MV to be the 2 CBs, while the RPV was labeled as NCB. This was consistent with the ablation responses in the clinic, as the roof ablation connected a CB to a NCB, leaving the other CB at the MV to take over the rhythm of the AT.

**FIGURE 9:**
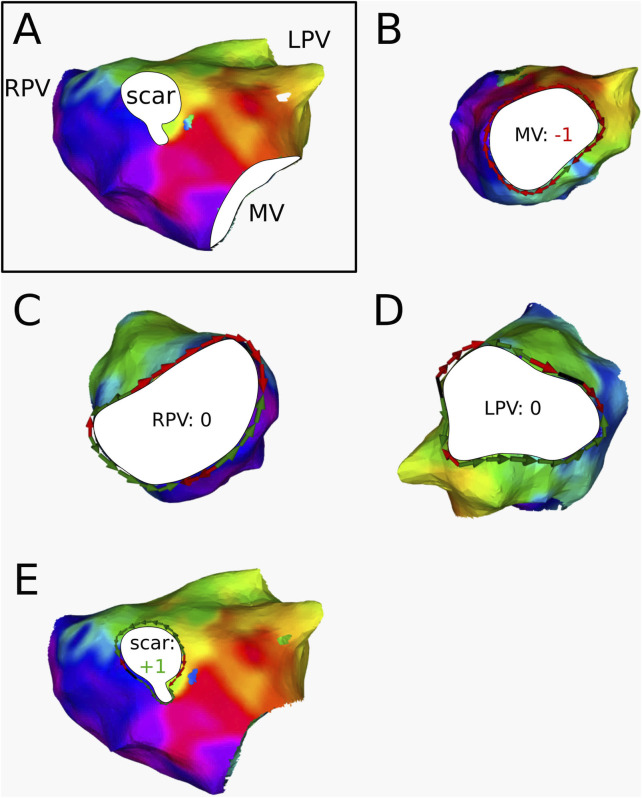
**(A)** Electroanatomical map of ${\text{AT}}_{3}$ in anterior-posterior (AP) view. The cyclical colors on the figure represent the LAT of the map. The boundaries are colored in white. **(B)** Conduction around the MV. The index was determined to be −1 **(C)** Conduction around the RPV. The index was determined to be 0 **(D)** Conduction around the LPV. The index was determined to be 0 **(E)** Conduction around the anterior scar. The index was calculated to be +1. This AT was clinically ablated with a line from the scar to the MV, resulting in termination.

${AT}_{3}$ (Figure 9) was determined to have 4 boundaries: the RPV, LPV, MV, and anterior scar tissue. The case was diagnosed as a double-loop reentry with a period of 260 ms and circuits around the MV and around the anterior scar. The ablation procedure interconnected the MV and the scar, thereby terminating the AT. DGM-TOP confirmed this diagnosis by identifying both MV and scar as CB, while the RPV and LPV were labeled as NCB.

#### 3.2.2 Boundary cycle length

**FIGURE 10:**
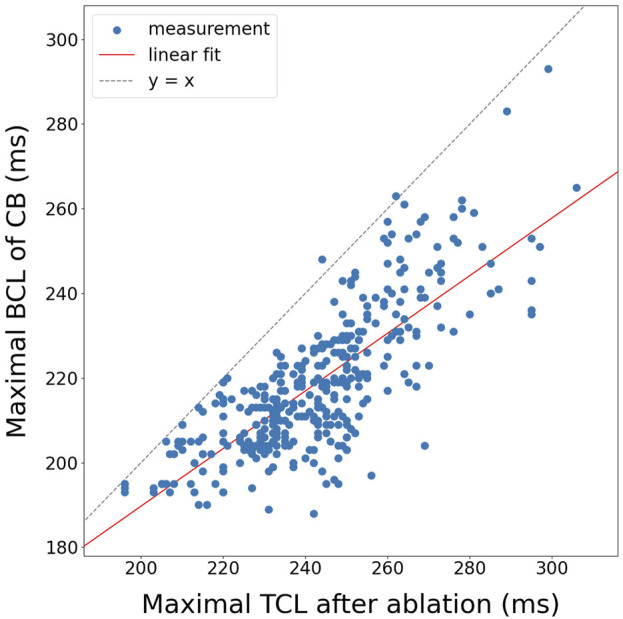
Correlation of the maximal BCL of a boundary to the maximal TCL after ablation for all 3B and 4B simulations (N = 378).

In 16 ATs, the CBs were interconnected by ablation, resulting in termination in all 16 cases. In the 8 other ATs, one CB was missed during diagnosis and subsequently was not ablated. Therefore, these ATs did not terminate; however, they changed to a slower AT. For these cases, the correlation of the BCL from the CB that was not ablated to the TCL of the AT after ablation was investigated. All results are represented in Table 3. A visual representation of the data is provided in the Supplementary Figure S6, formatted similarly to Figure 10.

**TABLE 3:** Calculated maximal BCL of the critical boundaries, compared to the TCL after ablation in each tachycardia that was not terminated after ablation.

${AT}_{i}$	${\text{AT}}_{1}$	${\text{AT}}_{2}$	${\text{AT}}_{4}$	${\text{AT}}_{11}$	${\text{AT}}_{14}$	${\text{AT}}_{18}$	${\text{AT}}_{19}$	${\text{AT}}_{24}$
TCL	285	230	265	260	335	290	270	295
${BCL}_{\max}$	342	330	351	322	376	494	332	454

Fitting a linear function through the data using linear regression, a positive correlation was achieved with a slope of 0.97±0.75 , an intercept of 105±212 ms, a coefficient of determination $\left(r^{2}\right)$ of 0.46 and a p-value of 0.248 as calculated by the Wald test. While a positive correlation is observed, the error and p-value are too large to draw statistical significant conclusions on this clinical data.

## 4 Discussion

The concept of the paired loops was initially introduced as a consequence of the index theorem in atrial tachycardia (AT) in Vandersickel et al. (2024). Concurrently, Santucci et al. (2023) proposed the term ‘critical boundaries’ (CBs). Both studies independently concluded that ablation between CBs reliably terminates AT. However, Santucci et al. provided a heuristic approach, lacking a rigorous mathematical foundation, compared to the al atopological approach in Duytschaever et al. (2024) to systematically identify CBs. In This research, these concepts were validated across 131 clinical cases and 572 simulations, demonstrating that the index theorem consistently confirms the presence of pairs of counter-rotating waves and identifies the optimal ablation line as the isthmus between CBs. Building on these findings, this manuscript presents a novel algorithm for the automatic calculation of indices and subsequent identification of CBs.

Previous research on ablation of ATs has suggested the existence of near-complete rotations (Takigawa et al., 2019; Kaiser et al., 2019). In these studies, the authors attempt to identify the optimal ablation line for AT based on theoretical ablation in schematic models. However, their approach appears more observational in nature, relying on trial and error by “theoretical ablation” methods, rather than a fundamental framework like the one used in DGM-TOP algorithm, which is grounded in the index theorem. Furthermore, a key advantage of this method is that it is fully automatic, streamlining the process for clinicians by eliminating the need for manual adjustments or subjective decision-making.

In Duytschaever et al. (2024), a method was discussed for determining the TCL of an AT after incorrect ablation by means of entrainment mapping. Here, it is stated that the maximal dPPI value around both CBs is positively correlated with the TCL-prolongation of the second AT. However, clinically these entrainment maneuvers are challenging and time-consuming. Therefore, in this manuscript, a method was proposed to determine the TCL after ablation without dPPI data by calculating the BCL of each CB and comparing them to the TCL after ablation.

In the simulations, using a basic formula (Equation 5), a moderate correlation $\left(r^{2}=0.62\right)$ was observed between the BCL and the TCL following incorrect ablation. Notably, the estimation consistently underestimates the TCL, likely due to the spherical geometry of the substrate, which increases the likelihood of wave collisions, perpendicular to the boundaries. This results in a reduced ΔLAT between sections in these regions, leading to an underestimation of the true BCL. For clinical cases, a $r^{2}$ value of 0.46 was observed, which is comparable to the *in silico* data, indicating a moderate correlation. However, the p-value is too high for statistical significance, which can be attributed to the small sample size (N = 8), limiting the statistical power of the analysis. Consequently, while the results are promising, no definitive conclusion can be drawn from this limited clinical dataset.

### 4.1 Clinical implications

As described in Arantes et al. (2011), transition to a second AT after the first ablation occurs frequently. In their study, the authors performed ablation on patients suffering from persistent AF. After this first ablation-procedure, the authors observed transition to AT and performed multiple subsequent ablations to reach termination. In total the authors noticed 39 AT-to-AT transitions. 35 of recorded ATs (89.74%) transformed into a slower AT or an AT with the same cycle length. This phenomenon was also described in Rostock et al. (2010). We therefore claim that these AT-to-AT transitions can be avoided by using DGM-TOP, identifying all CBs automatically and objectively, meanwhile minimizing ablated tissue and saving time by preventing the re-mapping of patients, therefore shortening procedure time and enhancing the understanding of the mechanism of AT.

### 4.2 Limitations

First, concerning the clinical data, boundary selection was made subjectively, based on the estimated location of non-conductive tissue. This most certainly induces a bias in the clinical dataset. While over- or underestimating the exact boundary edge will have minimal implications for the identification of the critical boundaries, the calculation of the BCL will be greatly affected. However, more critical is the identification of blocked lines. Missing certain blocked lines close to the boundary could possibly result in inaccurate arrows, resulting in a wrong TC calculation.

Second, at this moment, only a few clinical ATs (N = 8) with a TCL prolongation after ablation are observed. This problem occurs due to the lack of proper documentation concerning diagnosis, exact ablation lines, and ablation response. Therefore, a more extensive clinical study is needed to draw correct conclusions regarding the BCL-algorithm. However, at the moment the algorithm for TCL estimation offers only moderate correlation for simulations. The BCL-algorithm is currently very rudimentary and has room for improvement in the future.

### 4.3 Future research

Future research can focus on several key areas to enhance the algorithm’s robustness and clinical applicability. First, to eliminate subjectivity, an automated boundary-location algorithm leveraging voltage maps and signal processing techniques can be developed to identify fractionation and double potentials more reliably. Second, while a threshold value for determining lines of block was set in the preprocessing pipeline, it is likely an overfitting to this specific dataset; future work should explore adaptive or data-driven thresholding methods to improve generalizability. Third, a larger-scale clinical study is currently underway to address limitations in sample size and enhance statistical power. Fourth, an *in silico* study incorporating more realistic anatomical structures and electrophysiological remodeling could help bridge the gap between the simplified sphere-based model and clinical data. Fifth, the BCL-algorithm can be refined by modifying boundary path calculations to ensure the shortest possible trajectory, similar to a convex hull approach. Additionally, incorporating local conduction velocity measurements into the algorithm may further improve accuracy and predictive capability.

Topology has proven to be a powerful tool for offering novel insights into the underlying mechanisms of AT. Importantly, these same topological principles can be extended to other cardiac arrhythmias. Currently, we are exploring the application of DGM-TOP to bi-atrial tachycardia (Kitamura et al., 2018; Lai et al., 2023a; Lai et al., 2023b), ventricular tachycardia (Guandalini et al., 2019), both of which present complex topological structures and atrial fibrillation (Child et al., 2018; Wijesurendra and Casadei, 2019; Roy et al., 2020; Azzolin et al., 2023; Roney et al., 2020; Boyle et al., 2019), which exhibits substantially more complex electrophysiological mechanisms.

## 5 Conclusion

Topology and the index theorem serve as a highly effective tool for analyzing and diagnosing the driving mechanism of AT. Once the boundaries of AT are defined, DGM-TOP can automatically and objectively identify the CB that needs to be connected by an ablation line. Additionally, an algorithm was proposed to estimate the level of TCL prolongation after incorrect ablation by using the BCL of the CBs. However, this method needs to be refined to draw further conclusions. Additionally, more data is needed to draw statistically significant conclusions. Moreover, this topological approach and algorithm show significant potential for addressing more complex cardiac arrhythmias in the future.

## Data Availability

The data analyzed in this study is subject to the following licenses/restrictions: Simulations are to large to include, but are available on request. Clinical data can not be shared due to privacy. Requests to access these datasets should be directed to Nele Vandersickel, nele.vandersickel@ugent.be.

## Abbreviations

LA, left atrium; RA, right atrium; AT, atrial tachycardia; LPV, left pulmonary vein; RPV, right pulmonary veins; MV, mitral valve; SVC, superior vena cava; IVC, inferior vena cava; TV, tricuspid valve; TC, topological charge; CCW, counter-clockwise; CW, clockwise; TCL, tachycardia cycle length; LAT, local activation time; CB, critical boundary; NCB, non-critical boundary; BCL, boundary cycle length; SCZ, slow conductive zone; LOB, line of block.
