# Supplementary figures and images for: DGM-TOP: automatic identification of the critical boundaries in atrial tachycardia
Source: Front Physiol. 2025 May 27;16:1563807. doi: 10.3389/fphys.2025.1563807 (PMC12149188; doi:10.3389/fphys.2025.1563807)

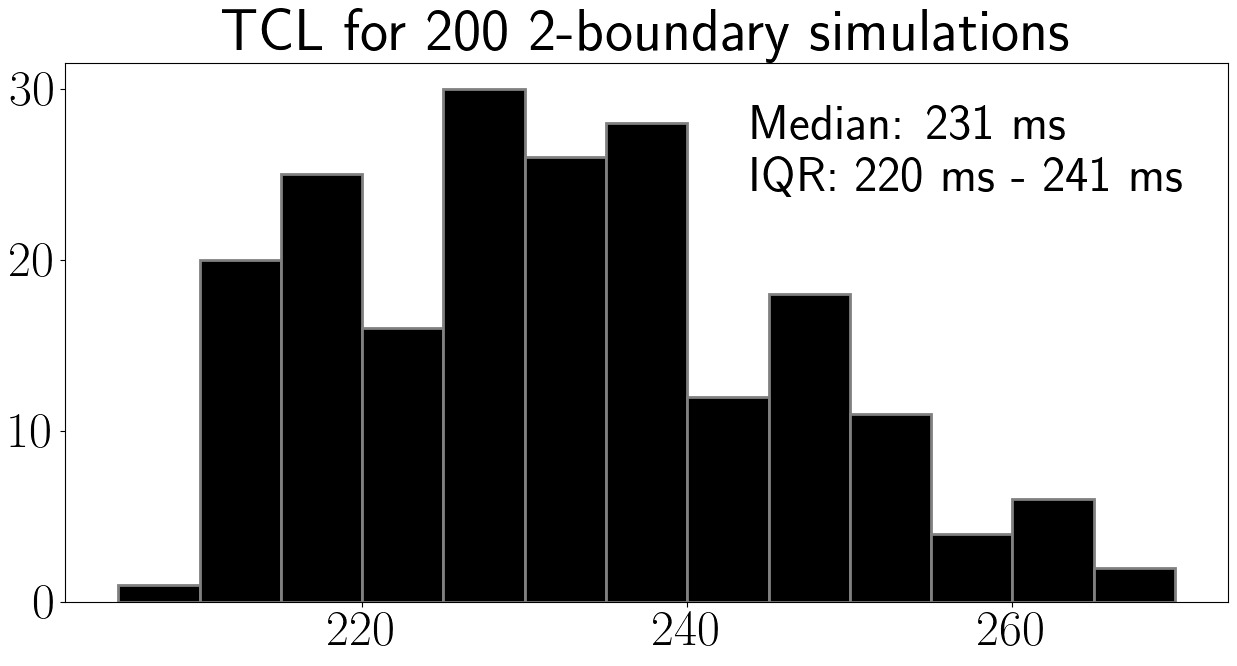

Supplement: Supplementary file 1 [file Image3.jpeg]

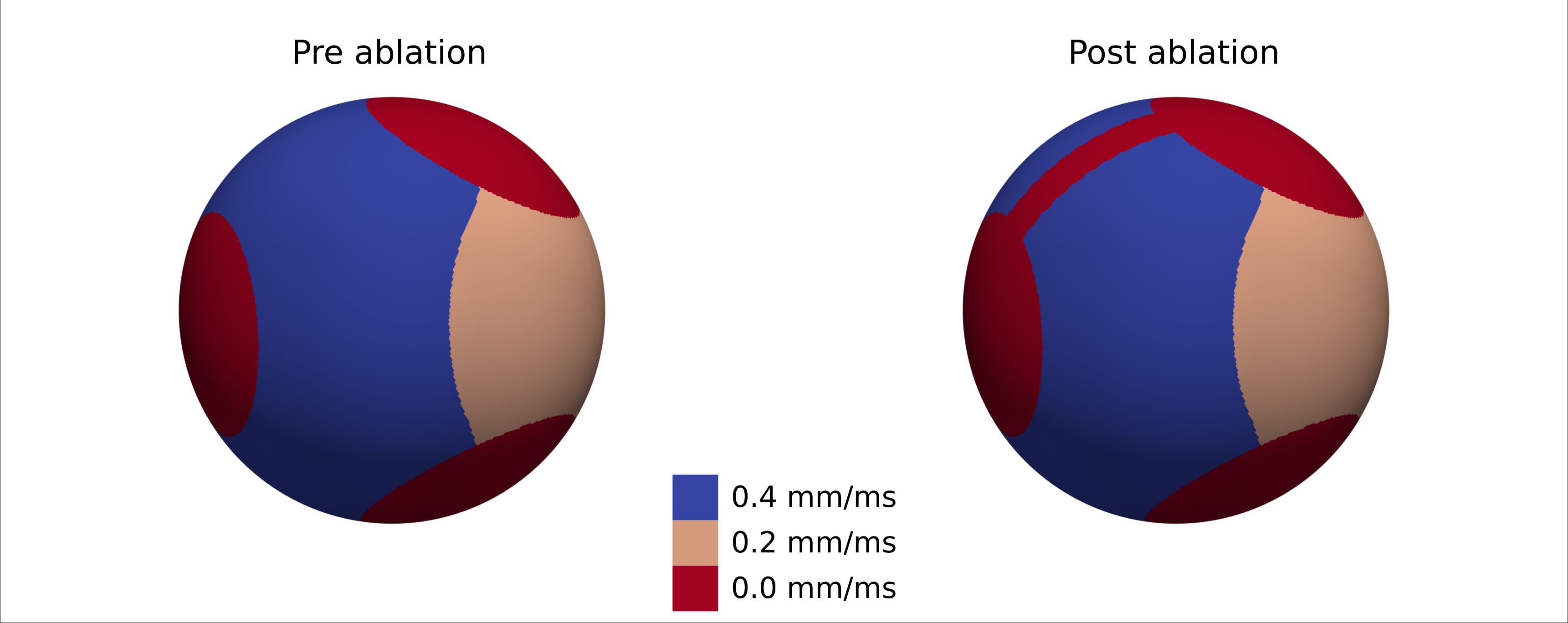

Supplement: Supplementary file 2 [file Image1.jpeg]

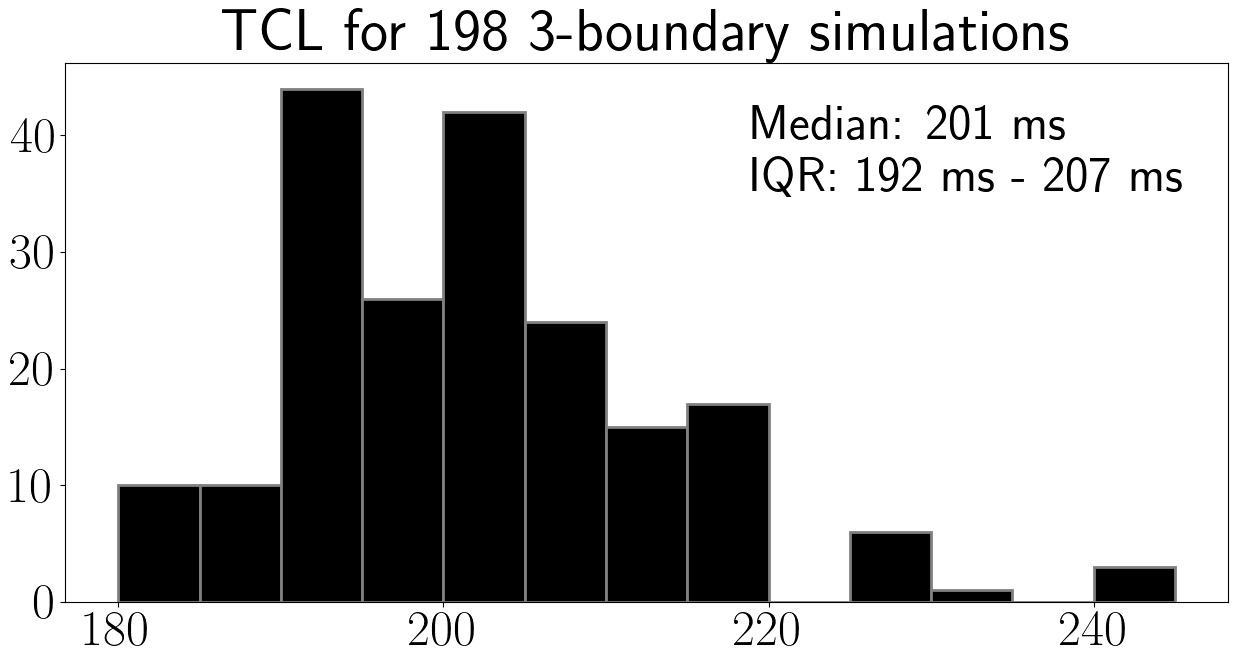

Supplement: Supplementary file 3 [file Image4.jpeg]

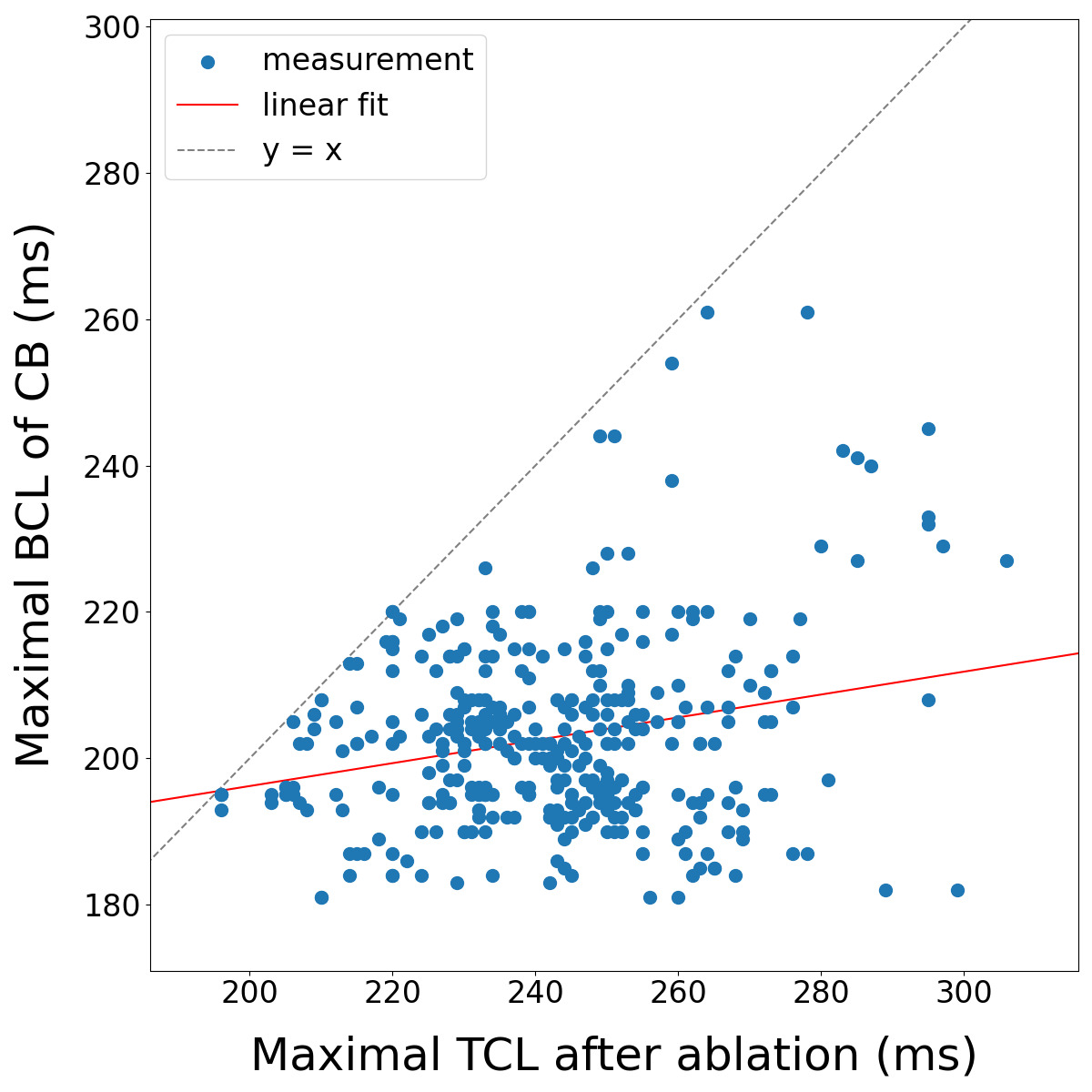

Supplement: Supplementary file 4 [file Image2.jpeg]

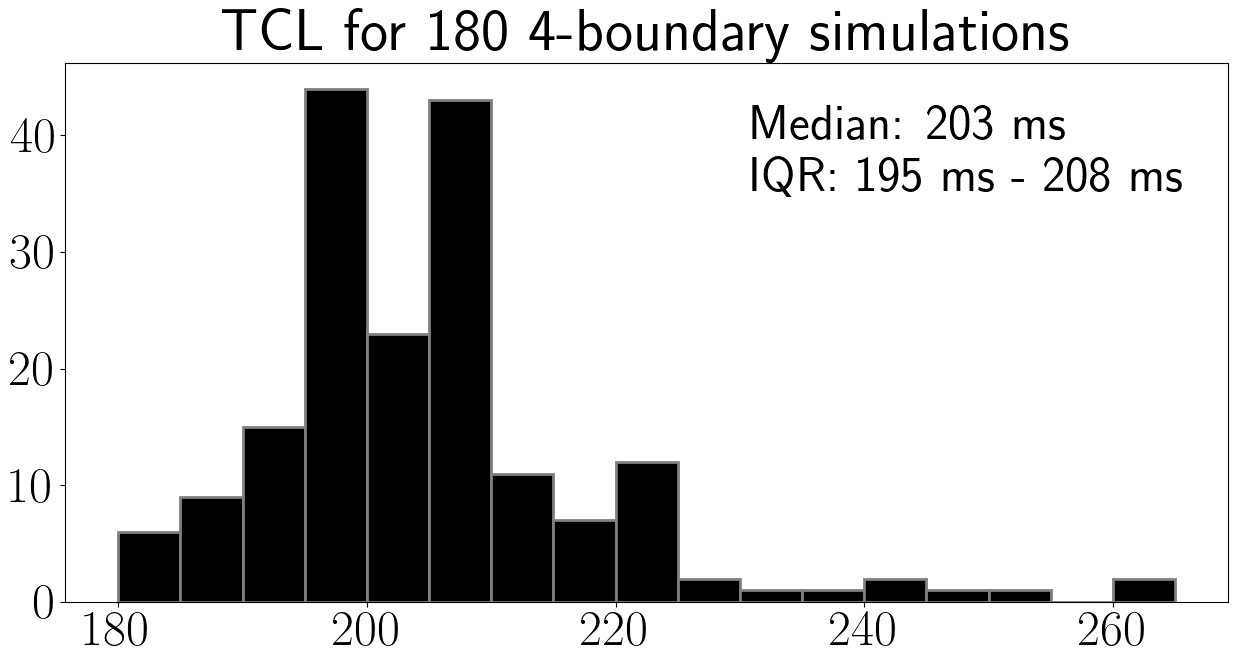

Supplement: Supplementary file 5 [file Image5.jpeg]

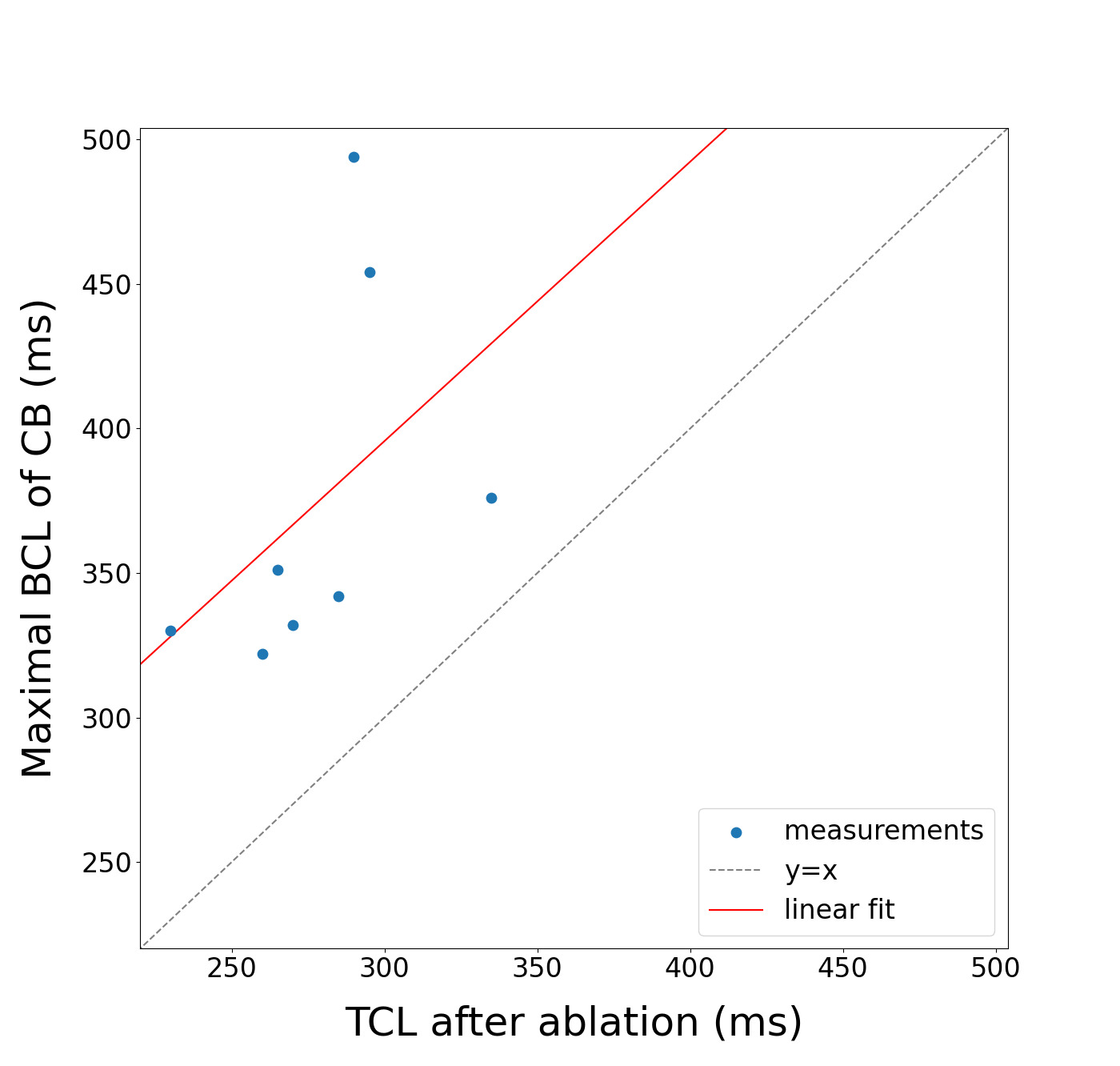

Supplement: Supplementary file 7 [file Image6.jpeg]
