# Supplementary material for: DGM-TOP: automatic identification of the critical boundaries in atrial tachycardia
Source: Front Physiol. 2025 May 27;16:1563807. doi: 10.3389/fphys.2025.1563807 (PMC12149188; doi:10.3389/fphys.2025.1563807)
Supplement: Supplementary file 6 [file DataSheet1.pdf]

6 Supplementary

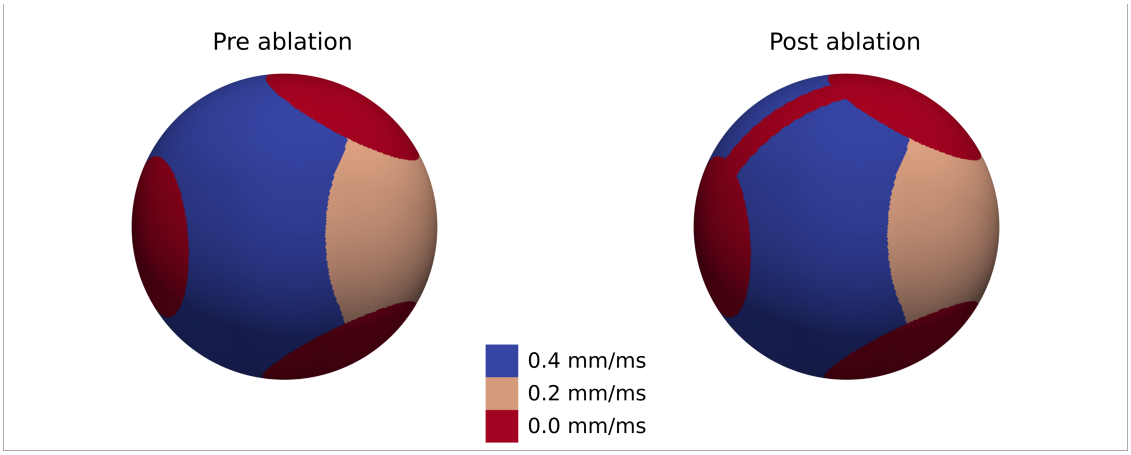

Figure S1: Example of a 3-boundary simulation substrate with corresponding conduction velocities. (Left) During stable AT-simulation (Right) After ablation

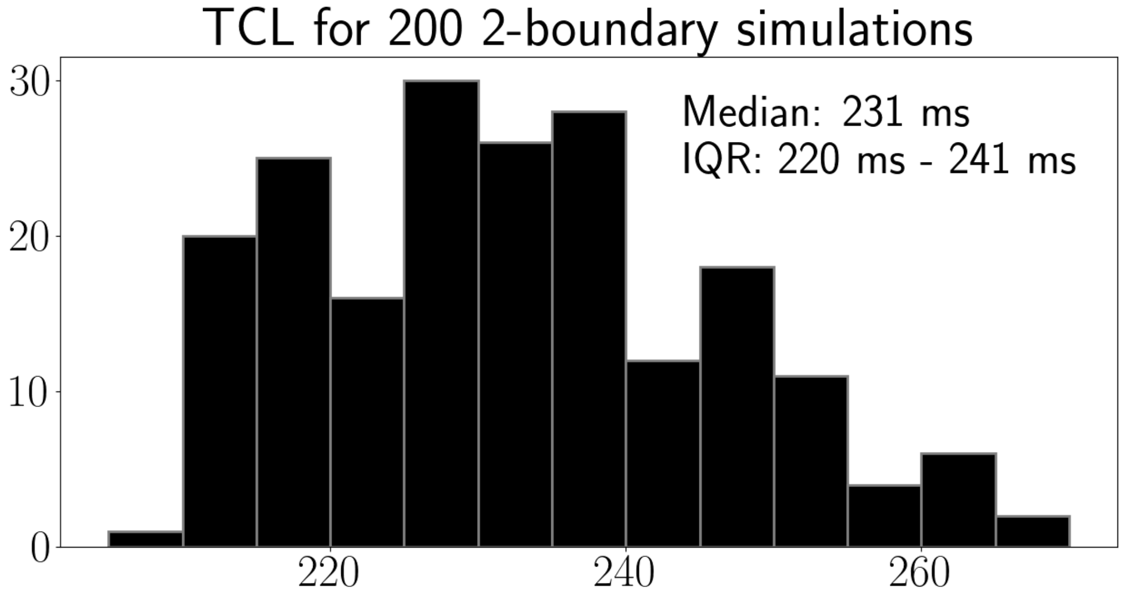

Figure S2: TCL distribution for *in-silico* MRAT with 2 boundaries. (N=200)

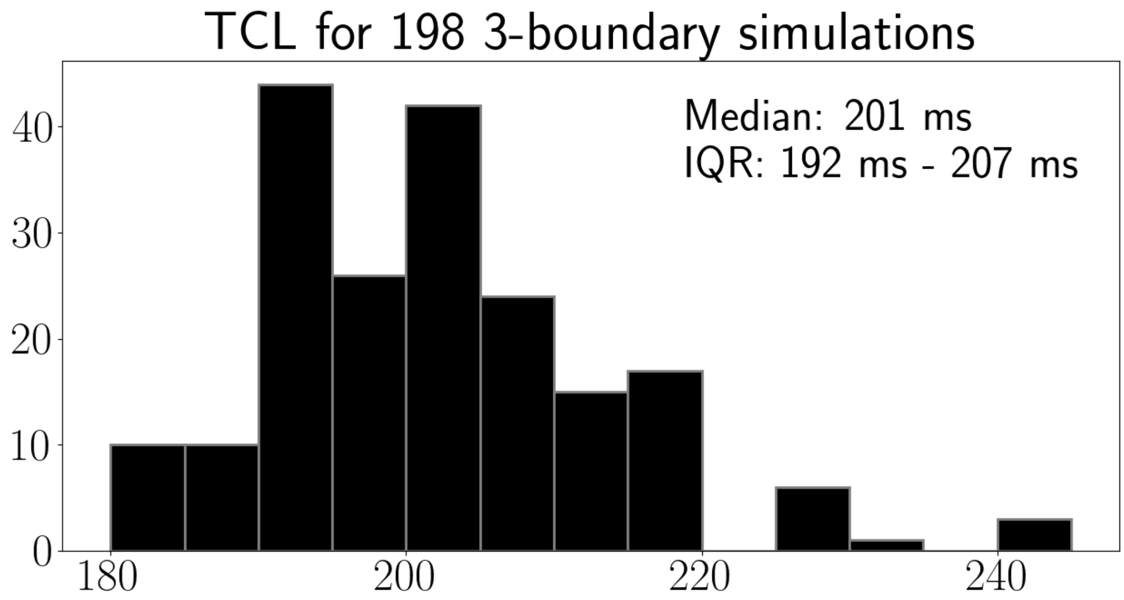

Figure S3: TCL distribution for *in-silico* MRAT with 3 boundaries. (N=198)

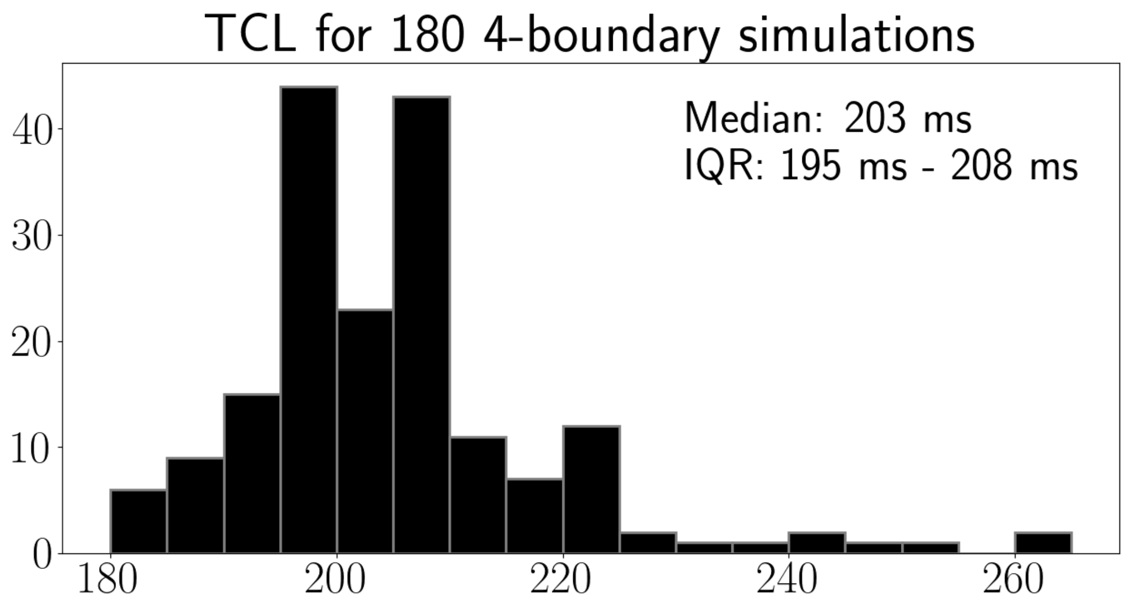

Figure S4: TCL distribution for *in-silico* MRAT with 4 boundaries. (N=180)

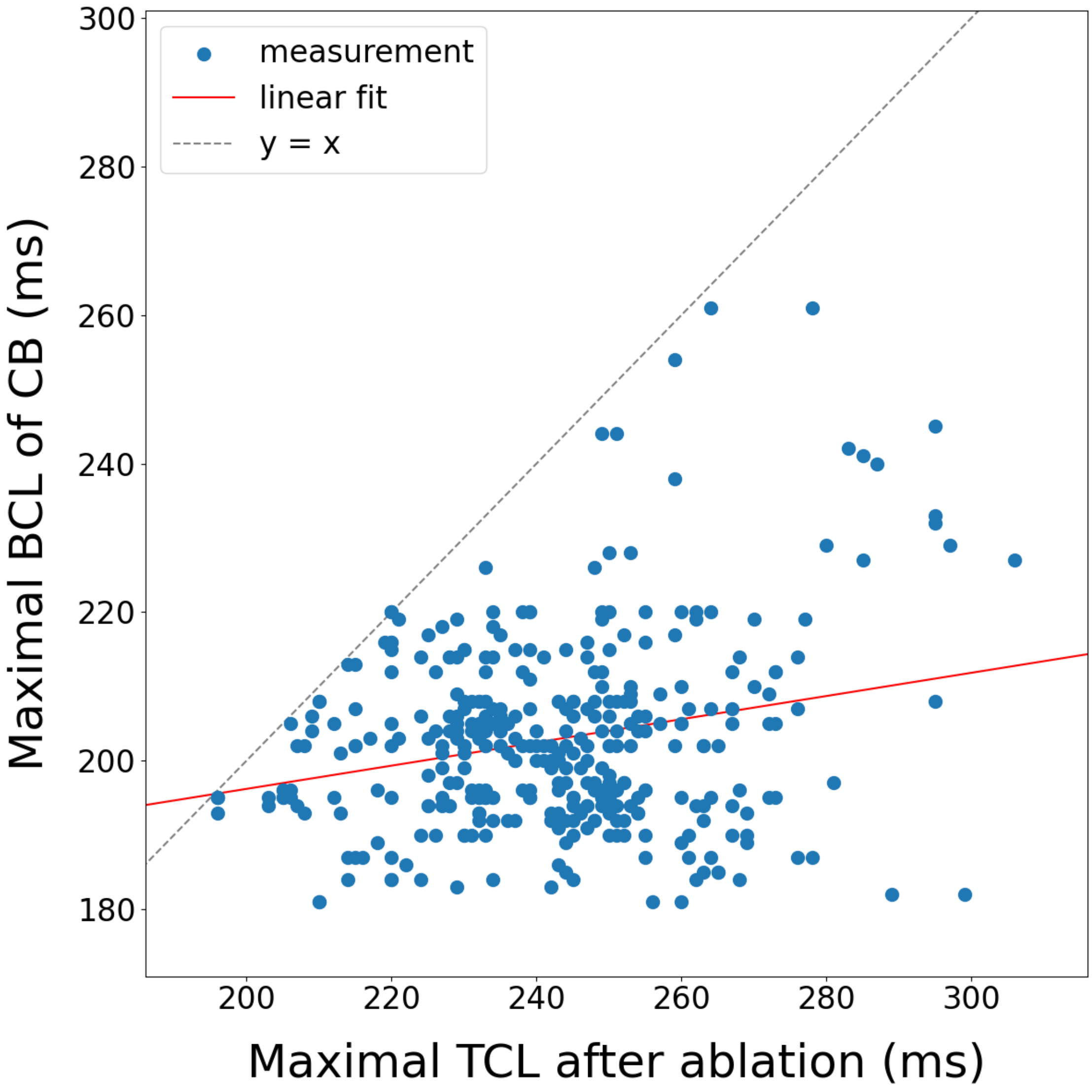

Figure S5: Correlation between Max TCL after ablation and the TCL of the stable state in simulated AT. no correlation is observed

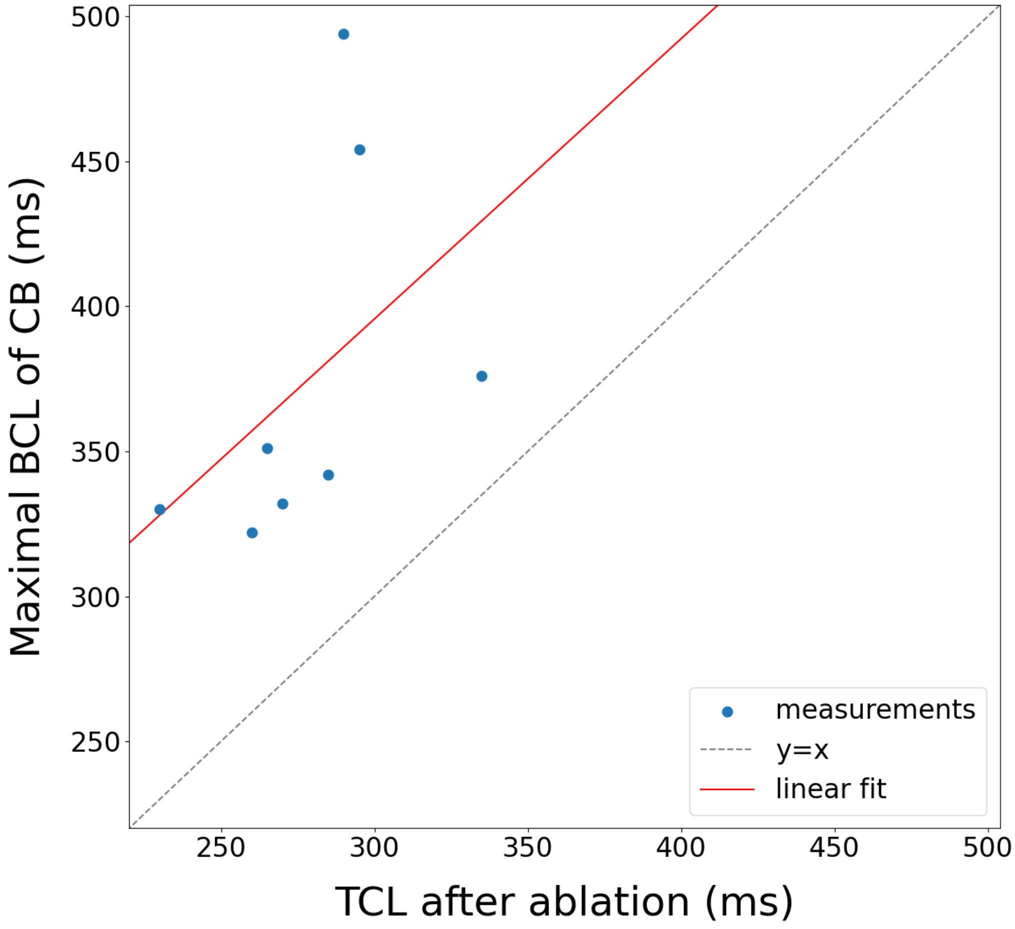

Figure S6: Correlation of the BCL of CBs that are not ablated to the TCL after ablation
